# Clinical outcome in elderly Chinese patients with primary hepatocellular carcinoma treated with percutaneous microwave coagulation therapy (PMCT): A Strobe-compliant observational study

**DOI:** 10.1097/MD.0000000000011618

**Published:** 2018-08-21

**Authors:** Xiaozhang Shen, Sicong Ma, Xiaoyin Tang, Tao Wang, Xingxing Qi, Jiachang Chi, Zhi Wang, Dan Cui, Yuan Zhang, Ping Li, Bo Zhai

**Affiliations:** Department of Interventional Oncology, Renji Hospital, School of Medicine, Shanghai Jiaotong University, Shanghai, China.

**Keywords:** age, disease progression, hepatocellular carcinoma, overall survival, percutaneous microwave coagulation therapy

## Abstract

Percutaneous microwave ablation therapy (PMCT) has been recommended for elderly hepatocellular carcinoma (HCC) patients who cannot tolerate surgery due to their age or presence of comorbidities. Few studies have investigated efficacy and treatment outcomes for PMCT treatment in these patients, especially in China, where patients are more often diagnosed and treated early in life. This study evaluated the safety and efficacy of ultrasound-guided PMCT in treatment-naive elderly HCC patients, and analyzed risk factors associated with poor treatment outcomes.

The 65 HCC patients in this retrospective study were divided into 2 groups: <65 years old or ≥65 years old. Patients received PMCT every month until tumor was unobservable and were then followed for 1 month after ablation. The primary clinical endpoint was the rate of complete tumor ablation, and secondary endpoints were progression-free survival and overall survival.

Patients ≥65 years old had significantly poorer performance status than younger patients, but similar rates of complete ablation. Multiple tumors and hypertension were associated with a significantly higher risk of death, while higher postoperative alanine aminotransferase levels were associated with a significantly lower risk of death. Patients with tumor sizes >5 to ≤ 10 cm were at a significantly higher risk for disease progression than patients with tumor sizes >1 to ≤ 3 cm. Complete ablation significantly lowered the risk of disease progression.

PMCT is safe and effective for patients ≥65 years of age, achieving total ablation in more than 90% of patients. Age and comorbidities did not affect clinical outcome.

## 1 Introduction

Hepatocellular carcinoma (HCC) is the third-most common cause of cancer-related death worldwide,^[1]^ and is the fifth most common malignancy in China.^[2]^ HCC is typically diagnosed in the mid to late stages, and a number of risk factors have been reported, including hepatitis B virus (HBV) infection, a condition commonly found in China,^[3,4]^ and metabolic syndrome and type 2 diabetes.^[5]^ Curative therapies such as surgical resection and orthotopic liver transplantation have been shown to be effective, but only around 20% of HCC patients are suitable candidates for these therapies,^[6]^ and therefore, a number of less invasive therapies have also been used for management.^[6–11]^

Percutaneous microwave coagulation therapy (PMCT) is one such therapy. It has previously been reported to improve the symptom severity score and quality of life in women with symptomatic uterine fibroids,^[12]^ and to be a safe and effective option to treat patients with inoperable nonsmall cell lung tumors.^[13]^ When used as an ablative treatment for primary HCC, it has been shown to induce coagulative necrosis in tumors with unfavorable locations and tumors >3 cm in diameter.^[14]^ During PMCT, tumor cells are killed by insertion of an ultrasound-guided ablation needle, which produces microwave radiation and induces coagulation of cellular proteins.^[15,16]^ PMCT is minimally invasive compared with conventional surgery and is easy to use for repeated treatment and therefore achieve a higher rate of complete ablation. For patients unwilling or unable to receive surgery, PMCT is a good alternative treatment option. The major advantages of ultrasound-guided PMCT include real-time monitoring, accurate guidance and targeting of the tumor, minimal tissue trauma and damage, and a higher safety and the ability to reach a larger target area than radiofrequency ablation.^[1,3]^ A previous recent report showed no significant difference in overall survival (OS) and progression-free survival (PFS) between HCC patients with portal hypertension who received PMCT and those who underwent surgical resection.^[17]^ HCC patients treated with PMCT also had a significantly better prognosis with less invasive tumors and fewer complications than patients who received laparoscopic resection.^[15]^

Although HCC is most often diagnosed in middle-aged and elderly populations who have multiple comorbidities, age is generally not an important factor in determining clinical management strategies. But it has been suggested that elderly patients may have a risk of either undertreatment because age alone has been used as the cut-off criterion, or overtreatment because possible complications due to the presence of comorbidities have not been fully considered.^[18]^ A recent study has reported that age was an independent risk factor predicting OS in HCC patients treated with thermal ablation,^[19]^ but radiofrequency and microwave ablation were lumped together in this analysis, and it is not known whether age is a risk factor when PMCT is considered alone. And although older age may be a risk factor, elderly patients with comorbidities might still derive significant benefit from less invasive techniques such as PMCT.^[20]^

In China, a high percentage of elderly patients are either not suitable (because of their physical condition) or not willing to receive surgery. Not much is known about the efficacy and safety of PMCT in elderly (≥65 years) Chinese patients who have “late-onset” HCC compared with Chinese patients who are diagnosed with HCC at younger ages. The major goal of this study was to evaluate the safety and efficacy of ultrasound-guided PMCT in treatment-naive older (≥65 years) patients. Patients at risk for poor treatment outcomes can be identified by the exploratory univariate risk factor analysis. The primary clinical endpoint was the rate of complete tumor ablation, and the secondary endpoints were PFS and OS.

## 2 Methods

### 2.1 Patients

Written informed consent was obtained from all study participants, and the study protocol was approved by the Institutional Review Board of the Renji Hospital, Shanghai Jiao Tong University School of Medicine. This retrospective study included a total of 65 patients who presented with HCC at the hospital from September 2010 to June 2016. Inclusion criteria were age >18 years (most patients were middle-aged or older due to the nature of HCC), a diagnosis of HCC with no other types of primary liver cancer, no more than 3 foci, and a tumor diameter less than 10 cm, and patients who received PMCT as initial therapy and had no prior chemotherapy or targeted therapy of any type. A complete medical history was available for all study participants. The diagnosis of liver cancer was based on the Chinese Guidelines for the Clinical Diagnosis and Staging of Primary Liver Cancer developed by the Professional Committee of Liver Cancer of Chinese Anti-Cancer Association. HCC diagnosis was confirmed using color Doppler ultrasonography, computed tomography (CT), magnetic resonance imaging (MRI), or pathological examination.

Patients were divided into 2 groups < 65 and “elderly” ≥65 years. The cut-off age of ≥65 years was used to define “elderly” according to the Organization for Economic Co-operation and Development definition of this age group.

### 2.2 Instruments

Ablation was performed under the guidance of Esaote Europe B.V. diagnostic ultrasound (Netherlands) using the FORSEA MTC3C microwave tumor therapy system (Nanjing Qinghai) with a microwave frequency of 2450 KHz and output power of 0∼150 W (the study used 80 W). The system could be adjusted continuously and had a cooled transmission cable and a 14G/15 cm microwave antenna treated with an anti-adhesion agent. There were 2 output electrodes for pulse output.

### 2.3 Procedures

#### 2.3.1 Pre-ablation preparation

Patients were subjected to routine blood tests, detection of liver, kidney and blood coagulation functions, detection of the blood lipid profile, detection of fasting blood glucose, electrocardiography, chest X ray, and detection of tumor-related markers before surgery. Patients fasted for 6 to 8 hours before surgery. Urinary catheterization was performed before surgery in patients with a tumor diameter >5.0 cm.

#### 2.3.2 Localization with ultrasound

Color Doppler ultrasound was performed soon after admission, in order to determine the site, size, shape, and number of tumors, internal echoes, and relationship with surrounding tissues. In addition, the pressure exerted by the tumor on the surrounding tissues and/or vessels, as well as presence of metastasis, were confirmed before the procedure.

#### 2.3.3 PCMT procedure

Patients were placed in a supine position, and the surgical site was disinfected. Patients received ondansetron before anesthesia in order to prevent vomiting. During surgery, 0.9% sodium chloride solution (500 mL; 1000 mL for dual needles) was used as cold circulating liquid. The tumor was punctured under ultrasound guidance using a 14G (diameter) × 180 mm (effective length) water-cooled microwave needle with microwave antenna. When the needle tip was 0.2 to 0.4 cm deeper than the bottom of the tumor, the water circulation system was switched on with an output power of 80 to 100 W for in situ tumor heating. Ablation time was determined on the basis of tumor size and the hyper-echo covering the tumor and tissues 1.0 cm away from the tumor during ablation. Ablation was generally performed for 3 to 8 minutes in patients receiving local anesthesia. In tumors with diameter < 3 cm, a single needle was used for puncture, and ablation was performed at one or more sites. In tumors with diameter >3 cm, dual needles were used for puncture, and ablation was performed at more than one site. When the tumor diameter was 5.0 cm or larger, blood volume was increased, 5% sodium bicarbonate was infused, urine pH was made basic, and furosemide was intravenously injected to ensure that the 24-hour urine volume was about 2000 mL. The color, nature, and volume of the urine, and the related kidney function were closely monitored. Damage to the intrahepatic vessels, bile ducts, gallbladder, gastrointestinal tract, kidney, diaphragm, lung, pericardium, and important tissues in the hepatic hilum were avoided during the intraoperative puncture. After microwave ablation, the output power was reduced to 70 W, and heating was done for about 5 seconds during each retraction of the needle to avoid implantation of tumor cells or hemorrhage. After treatment, the wound was focally dressed. Patients who received local anesthesia were transferred to the ward when their condition stabilized; patients who received general anesthesia were transferred to the recovery room and then to the ward when they became completely conscious. Oxygen supplementation and vital signs were closely monitored. Anti-infection therapy, hemostasis, hepatoprotective therapy, and analgesia were also administered.

#### 2.3.4 Frequency of ablation

Ablation was done once every month until the tumor was completely unobservable. Patients were followed up for about 1 month after the procedure, and evaluation of the tumor was performed after each ablation.

For tumors with a diameter ≤5 cm, pre-surgery planning was for 1 ablation. For tumors with a diameter >5 cm, pre-surgery planning was for 2 ablations. The time interval between the 2 initially planned ablations was 21 to 30 days, and this was dependent on the postoperative recovery. Data collected after each ablation were analyzed. Dynamic CTs or MRIs were used to confirm whether complete ablation was achieved.

#### 2.3.5 Precautions for surgical procedures

##### 2.3.5.1 Selection of route for intervention

The use of the optimal, correct route for intervention reduced the puncture distance, avoided major vessels, minimized damage to normal tissues, increased puncture accuracy, and reduced complications.

##### 2.3.5.2 Contraindications

The contraindications are severe liver dysfunction or hepatocellular jaundice, evidence of severe ascites with mild oliguria that improved after hepatoprotective and diuretic therapies, evidence of kidney dysfunction, evidence of coagulation dysfunction or bleeding tendency, severe hypertension, coronary heart disease, or cardiac dysfunction, tumor volume >60% to 70% of the liver; extrahepatic or diffuse metastasis; end-stage liver disease; and presence of systemic or focal acute or active infection. When there was complete obstruction of the portal vein by the tumor, the decision for surgical intervention was made depending on the collateral vessels of the portal vein, tumor size, and the severity of the esophageal varices.

##### 2.3.5.3 Statistical analysis

Mean, standard deviation (SD), and range were calculated for data on age, preoperative aspartate aminotransferase (AST), alanine aminotransferase (ALT), and HBV DNA levels, tested by nonparametric Mann–Whitney *U* test. Frequency and percentages were computed for categorical variables, tested by Chi-square test or Fisher exact tests. Univariate Cox proportional hazard model was used to determine the effectors of poor survival and disease progression. Multivariate Cox proportional hazard model was not performed due to the limited sample size. We evaluated the effect of variables such as multiple tumors, hypertension, postoperative ALT levels, age, sex, smoking, drinking, tumor size, hepatitis, Child–Pugh classification, alpha fetoprotein (AFP), cancer antigen 19–9 (CA19–9), AST, HBVDNA, type 2 diabetes mellitus, cardiovascular disease, and type of ablation on OS and PFS. The strength of the relationship between an independent variable and OS or PFS was quantified with the hazard ratio (HR); the HR's 95% confidence interval (95% CI) was also estimated. The duration of OS was calculated from the date of surgery until the date of death or the last follow-up visit. Similarly, the duration of PFS referred to the time interval between the date of surgery until the date of disease progression or the last follow-up. A *P* value < .05 was considered statistically significant. All statistical analyses were 2-sided and used PASW software (version 21; IBM Corp., Armonk, NY).

## 3 Results

### 3.1 Patient characteristics

The study included a total of 65 HCC patients who had received no treatment before PMCT. The study population included 50 males (76.9%) and the mean age was 67.4 ± 6.5 years. Most of the study participants had a history of no smoking (87.7%) and no drinking alcohol (92.3%). Most of the study participants were also classified as Child–Pugh grade A (86.2%), had only a single tumor (72.3%), and had hepatitis (76.9%). The preoperative AFP was ≤20 ng/mL in 56.9% of study participants, and the preoperative CA19–9 was ≤50 U/mL in 72.3% of study participants. Sixty-one (93.8%) patients received complete ablation. The mean disease duration was 184.3 (SD = 133.7, range: 1–420) months. The mean AST and ALT levels were 60.5 (SD = 65, range: 13–438) U/L, and 289 (SD = 207.4, range: 34–1362) U/mL, respectively.

Patients were followed up for an average of 23.5 ± 14 months (range: 3–54). The mean duration of PFS was 12.4 ± 11.4 months (range: 1–51). In patients with recurrence, the average time to death after recurrence was 16.4 ± 11.4 (range: 2–46) months (data not shown).

### 3.2 Comparisons between patients < 65 years of age and ≥65 years of age

Study subjects were classified into 2 groups based on age (<65 and ≥65 years) and the clinical characteristics of the 2 groups of patients are summarized in Table 1. There were no significant differences between the 2 groups, except that the older age group was in worse general health in that they had poorer ECOG/WHO/Zubrod scores. The older group also had a higher percentage with type 2 diabetes, hypertension, and cardiovascular disease, but these differences did not reach statistical significance. Tumor characteristics, success in ablation, and clinical outcomes were not significantly different between the groups.

**Table 1:**
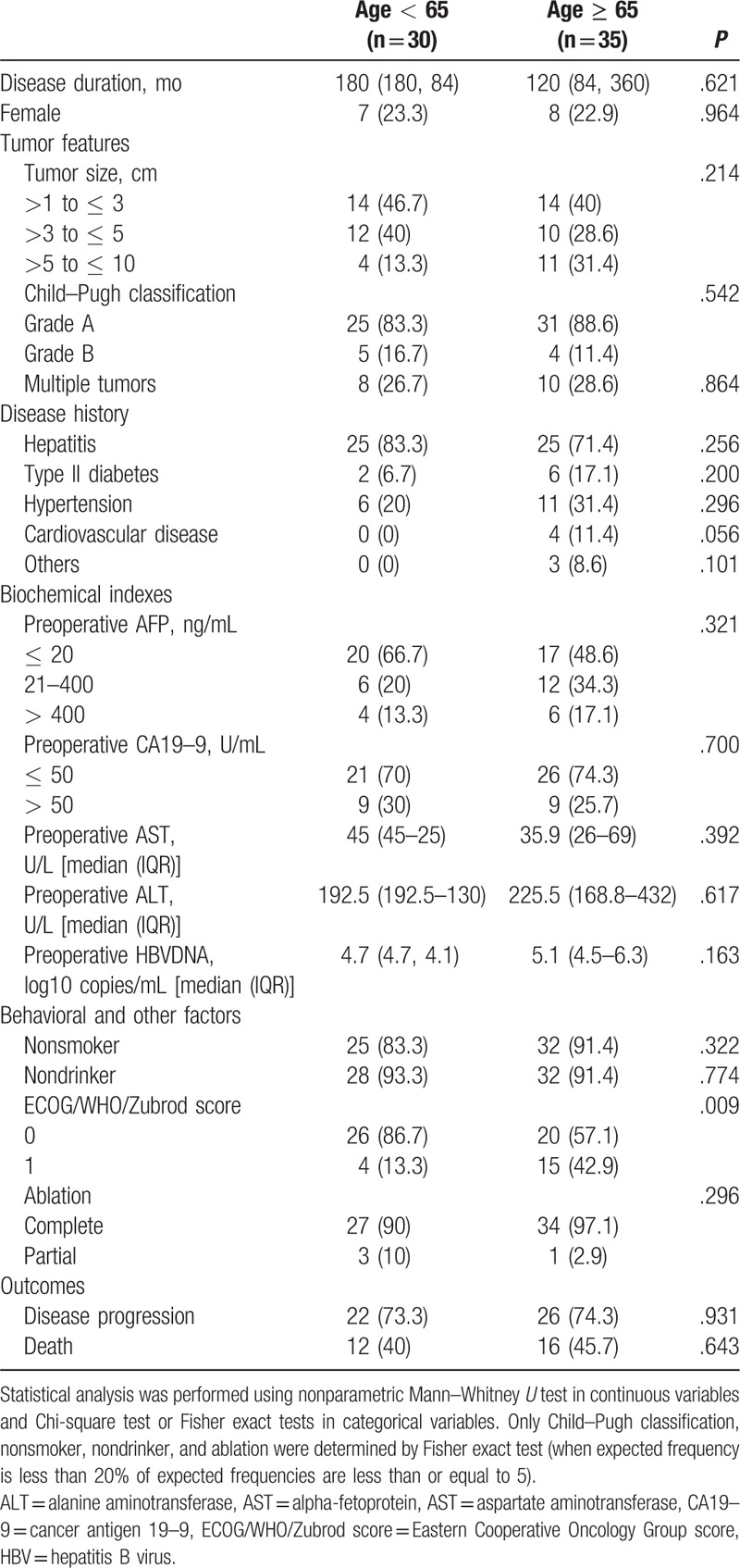
Patients’ characteristics and ablation rate (n = 65).

The < 65 age group had a disease duration of 180 months, while the ≥65 age group had a disease duration of 120 months. One patient had a pleural effusion and 2 patients had ascites after PMCT. There were no deaths after PMCT.

### 3.3 Effectors of poor overall survival

Twenty-eight deaths occurred during the follow-up period. Variables related to poor OS are summarized in Table 2. Patients with multiple tumors or hypertension had more than twice the risk of death compared with patients without these conditions. In contrast, patients with a higher postoperative ALT change had a slight, but significantly lower risk of death than patients with smaller changes in ALT levels. Age, tumor size, degree of ablation, and other patient characteristics had no significant effect on mortality. Factors such as gender, smoking, drinking, hepatitis, or Child–Pugh classification also had no effect on survival.

**Table 2:**
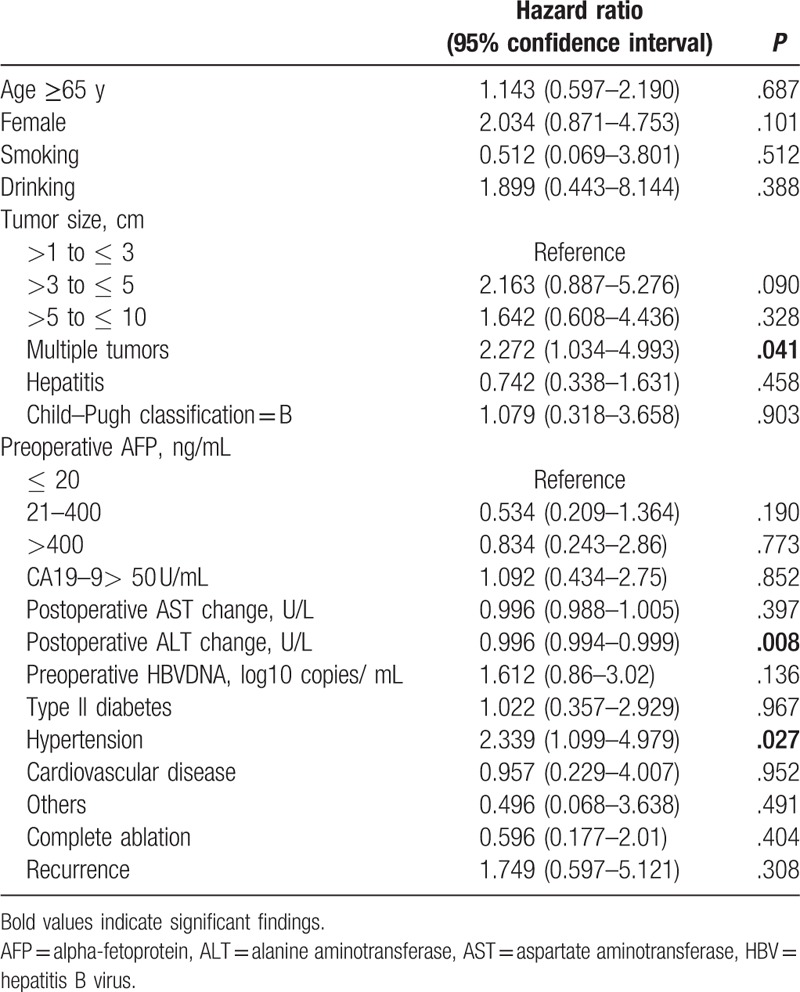
Factors affecting overall survival in 65 patients.

### 3.4 Effectors of progression-free survival

Forty-eight instances of disease progression occurred during the follow-up period, and the analysis of variables related to PFS are summarized in Table 3. Patients with tumor sizes of >5 to ≤ 10 cm were twice the risk for disease progression compared with patients with tumor sizes >1 to ≤ 3 cm. And, complete ablation significantly lowered the risk of disease progression. Comorbid conditions, Child–Pugh index, and biochemical parameters were not related to disease progression.

**Table 3:**
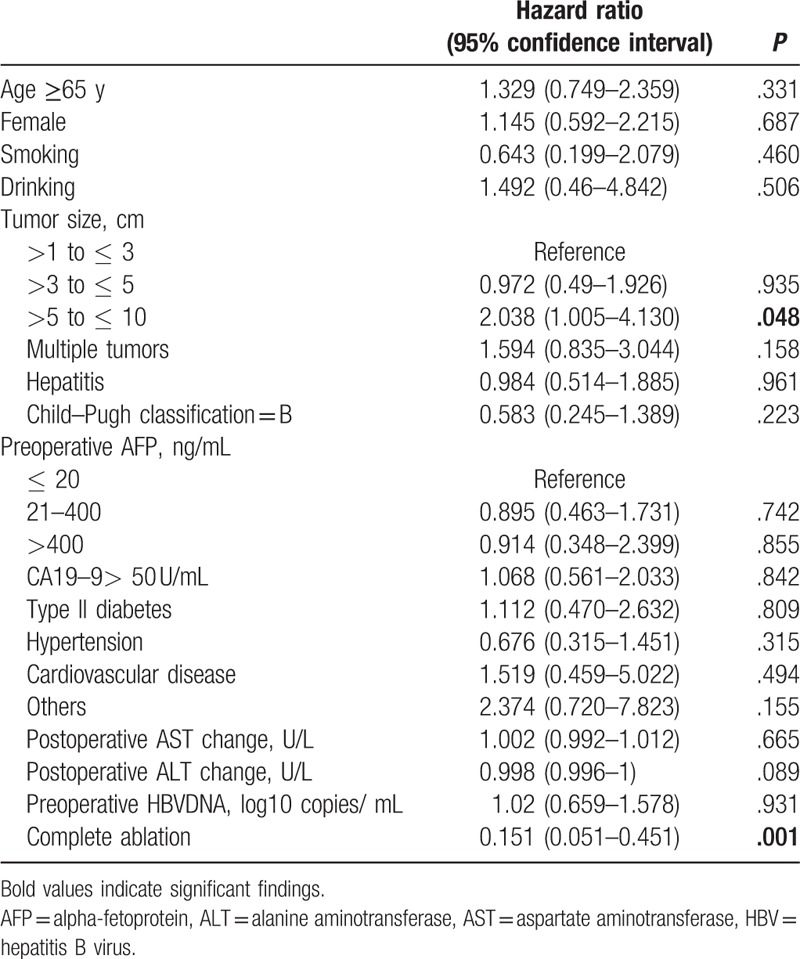
Factors affecting progression-free survival in 65 patients.

## 4 Discussion

This retrospective study investigated the safety and efficacy of ultrasound-guided PMCT in 65 treatment-naive HCC patients and evaluated variables that influenced OS and PFS in these patients. The elderly, ≥65, age group had a significantly poorer performance status than the < 65 age group, but did not differ in other characteristics. Older age was not a predictor of a higher risk of either death or disease progression. Hypertension, multiple tumors, and lower postoperative ALT levels were predictors of a higher risk of death, and tumor size and incompleteness of ablation were predictors of a higher risk of disease progression.

The current study included PMCT treatment of all tumors < 10 cm diameter. A previous comparison of PMCT treatment and surgical resection in HCC patients reported surgical resection to be the best option for treating patients with single, smaller (<3 cm diameter) tumors and PMCT to be a better choice for elderly HCC patients with comorbidities who were unable to tolerate surgery, as well as for HCC patients with portal hypertension, and for tumors located deep in the liver.^[17]^ There has been a general trend toward less frequent use of aggressive strategies such as surgical resection, and more frequent use of less aggressive treatments for elderly patients.^[21]^ When 2 less aggressive treatments, microwave ablation (the treatment used here) and radiofrequency ablation were used for HCC patients with multiple tumors, although both treatments damaged tumor cells by the same mechanism (thermal injury), microwave ablation had a more predictable ablation zone, caused more rapid coagulation,^[3]^ and resulted in lower rates of tumor progression than radiofrequency ablation.^[22]^

The incidence of HCC in the elderly population has been increasing.^[18]^ In China, this has been due partly to the progressive aging of the general population and partly to implementation of widespread vaccination and surveillance programs that have resulted in a significant increase in early-stage diagnosis of HCC. However, few studies have investigated the efficacy and outcomes of therapeutic strategies such as PMCT for patients who have HCC onset during later life, studies especially relevant for China, where most patients are diagnosed earlier in life.

Our study showed no relationship between age and clinical outcome after PMCT treatment of HCC. Clinical management of HCC among elderly patients is influenced by factors such as increased life expectancy, presence of comorbidities, and the benefit-to-risk ratio of specific treatment strategies.^[18]^ In studies of surgical resection for HCC, elderly patients had similar rates of OS and PFS compared with younger patients, although the older patients had more postoperative complications and a longer hospital stay than younger patients.^[23,24]^ Studies evaluating efficacy and safety of radiofrequency ablation in patients >75 years old have shown no significant difference in cumulative OS and relapse-free survival compared with those < 75 years old.^[25,26]^ However, other studies of radiofrequency ablation have shown elderly patients to have worse outcomes.^[26,27]^ A recent study comparing elderly (>70 years old) and younger (<70 years old) HCC patients treated with radioembolization reported no significant difference in outcome between the 2 groups.^[28]^ And a study of 192 HCC patients with an age range of 22 to 86 years found age to be independently associated with poor OS.^[29]^ And although the relationship between age and outcome for other HCC treatments has been studied, clinical outcomes of elderly patients receiving PMCT have not previously been reported, and our data showed that patients ≥65 years of age had similar clinical outcomes to those < 65 years of age.

And when the data from the 2 groups were combined for analysis, age did not increase the HR either for death or for a lower risk of PFS.

Tumor size was a predictor of OS, but not of PFS in our study. Tumor size has been reported to be an independent predictor of OS in HCC patients treated with a combination of transarterial chemotherapy and microwave ablation and in those treated with microwave ablation alone.^[29,30]^ In our study, incomplete tumor ablation was a significant predictor of low risk of PFS. Incomplete tumor ablation was also reported to be an independent unfavorable prognostic factor in HCC patients treated with percutaneous thermal ablation therapy.^[31]^ A study on 258 treatment-naive HCC patients treated with radiofrequency ablation previously showed that age >65 years, serum albumin levels < 3.7 g/dL, international normalized ratio (INR) >1.1, and α-fetoprotein >20 ng/mL were among the independent risk factors associated with poor OS, while age >65 years, multiple tumors, and tumor size were among the most important risk factors associated with recurrence.^[26]^ However, to the best of our knowledge, there are no data describing prognostic factors in a study cohort of elderly HCC patients receiving PMCT.

The current study is unique because it focuses on HCC patients >60 years old who were treated with PMCT. In this patient population, multiple tumors were associated with a higher risk of mortality, while a larger postoperative decrease in ALT levels was associated with a lower risk of mortality. Tumor sizes >3 to ≤ 5 cm predicted a higher risk of disease progression than tumor sizes >1 to ≤ 3 cm. And, complete ablation of the tumor was associated with a lower risk of disease progression. These results are consistent with previous data showing that patients with tumors >5 cm had a significantly lower likelihood of achieving initial complete ablation and had significantly lower PFS.^[32,33]^

The present study that focused only on elderly HCC patients expands our understanding of factors that predict the outcome of PMCT in this population of patients. No significant difference in the rate of complete ablation or in outcomes was observed between younger and older HCC patients, suggesting that PMCT is as suitable for older HCC patients with worse performance status as for younger patients. This information gives guidance for the clinical management of elderly HCC patients who cannot undergo curative treatment such as liver transplantation or surgical resection. The major limitation of our study was its retrospective nature, which limits its ability to predict risk factors. The risk factors found in the current study therefore need to be validated in a prospective study with a larger sample size.

## 5 Conclusion

PMCT is safe and effective for patients >65 years of age, achieving total ablation in more than 90% of patients. Age and comorbidities did not affect clinical outcome.

## Acknowledgment

The authors also would like to thank Ding Min for revision of the manuscript.

## Author contributions

**Conceptualization:** Jiachang Chi, Zhi Wang.

**Data curation:** Xiaozhang Shen, Sicong Ma, Yuan Zhang.

**Formal analysis:** Xiaoyin Tang.

**Funding acquisition:** Bo Zhai.

**Investigation:** Xingxing Qi.

**Methodology:** Sicong Ma, Dan Cui.

**Project administration:** Bo Zhai.

**Software:** Xiaoyin Tang.

**Supervision:** Jiachang Chi, Ping Li.

**Validation:** Tao Wang, Zhi Wang.

**Visualization:** Tao Wang, Zhi Wang.

**Writing – original draft:** Xiaozhang Shen.

**Writing – review & editing:** Tao Wang, Xingxing Qi, Jiachang Chi, Zhi Wang, Dan Cui, Ping Li, Bo Zhai.
